# Semi-Supervised Morphosyntactic Classification of Old Icelandic

**DOI:** 10.1371/journal.pone.0102366

**Published:** 2014-07-16

**Authors:** Kryztof Urban, Timothy R. Tangherlini, Aurelijus Vijūnas, Peter M. Broadwell

**Affiliations:** 1 The Scandinavian Section, University of California Los Angeles, Los Angeles, California, United States of America; 2 Department of English, National Kaohsiung Normal University, Kaohsiung, Republic of China; 3 The University Library, University of California Los Angeles, Los Angeles, California, United States of America; Stony Brook University, United States of America

## Abstract

We present IceMorph, a semi-supervised morphosyntactic analyzer of Old Icelandic. In addition to machine-read corpora and dictionaries, it applies a small set of declension prototypes to map corpus words to dictionary entries. A web-based GUI allows expert users to modify and augment data through an online process. A machine learning module incorporates prototype data, edit-distance metrics, and expert feedback to continuously update part-of-speech and morphosyntactic classification. An advantage of the analyzer is its ability to achieve competitive classification accuracy with minimum training data.

## Introduction

IceMorph [1] is a semi-supervised part-of-speech (POS) and morphosyntactic (MS) tagger for Old Icelandic. Old Icelandic is a difficult language to tag for morphosyntactic features given its inflectional and morphonological complexity. IceMorph is designed to achieve competitive classification accuracy using a minimum of cleanly tagged training data, and to allow for continuous online retraining.

The IceMorph system consists of a number of interacting modules, including a Perl machine parser for Old Icelandic dictionaries, a prototype-based inflection generator coded in Haskell based on similar tools used in Functional Morphology [11], [12], [22], an edit distance classifier, a website to collect feedback from human experts, and a context-based machine learning algorithm for grammatical disambiguation. We hypothesize that this multi-pronged approach can offer better outcomes than any one of the approaches alone to the vexing problem of morphological analysis in Old Icelandic. Although this may seem to be an obvious solution for the problem of POS and MS tagging in a language that not only has a complex morphology but also for which there is a paucity of clean training data and a noisy target corpus, we have not encountered similar multi-pronged approaches to this problem for Old Icelandic.

For the machine learning component, we rely on a Hidden Markov Model (HMM) classifier that makes use of the restricted Viterbi algorithm, and retrain from expert input as opposed to co-training [28]. Although recent work on sequential tagging has returned excellent results with Conditional Random Fields (CRF) [27], because of problems associated with Old Icelandic's inflectional complexity and the very limited scope of our training data, the CRF we implemented returned sub-optimal results. Instead, our results show that the multi-pronged approach we describe, despite a very small and noisy training set, can achieve competitive classification (96.84% on the POS task, and 84.21% on the MS task).

We took inspiration for IceMorph from a number of sources. Several tools exist for morphosyntactic tagging of Modern Icelandic; for instance [21], achieves 91.18% accuracy by applying a TnT tagger trained on an extensive corpus of Old Icelandic texts orthographically and grammatically normalized to Modern Icelandic. Another approach is IceTagger [23], a rule-based POS tagger for Modern Icelandic that achieves a 91.54% accuracy rate on a POS classification task. There are also a large number of semi-supervised Bayesian POS taggers such as [24], [25], with [24] reporting an accuracy of 79.7% on an MS classification task, and [25] reporting 93.4% accuracy on a POS task. However, all of the existing approaches require either a set of manually crafted rules or fairly extensive training sets. Importantly, the approaches for Icelandic described elsewhere [21], [23], [29] are all tuned for Modern Icelandic, a space in which relatively large, clean training data exist. A philosophical underpinning of IceMorph is to provide competitive tagging performance for Old Icelandic utilizing available resources while requiring a minimum of clean input data. For example, our training sets are an order of magnitude smaller than those used in [21]. Consequently, we feel that IceMorph is closely related to projects such as [5], [6], [29] which make use of language tools to reduce the amount of man-hours required to tag a corpus. [5] reports an accuracy of 93.1% on a Spanish POS task [6], reports an accuracy of 90.7% on a POS task in English, and [29] reports an accuracy of 93.84% on a POS task in Modern Icelandic (Table 1).

**Table 1:** Accuracies for different POS/MS taggers with commonalities to IceMorph.

Approach	POS classification	MS classification
IceMorph HMM-rV (Expert/Gold)	96.84%/73.16%	84.21%/54.86%
Loftsson [29]	93.84%	–
Cucerzan & Yarowsky [5]	93.1% (Sp)/89.2% (Ro)	–
Rögnvaldsson TnT [21]	91.8%	–
Loftsson IceTagger [23]	91.54%	–
Brill & Marcus [6]	90.7%	–
Feldman & Hana [24]	–	79.7%

For comparison, the accuracy of the IceMorph HMM-rV tagger is presented in the first row. Our measures of accuracy reflect the use of two distinct sets of tagged data. The first set (called EXPERT) contains longer sequences of training data and thus reflects more accurately IceMorph's performance when trained with a rich data set, and is also more comparable to the training data used in these comparison studies.

## Methods

### System architecture

IceMorph consists of a collection of modules designed to streamline the creation, maintenance, and analysis of input data as well as the prediction of POS and morphosyntactic (MS) classes for previously unseen words. It can be conceptualized as consisting of two separate systems. The first system produces an initial set of tags for each corpus instance, providing broad coverage (>98%) with sub-optimal accuracy. The second system refines the initial set of tags by continuously directing novel expert feedback into a machine learning algorithm.

**Figure 1:**
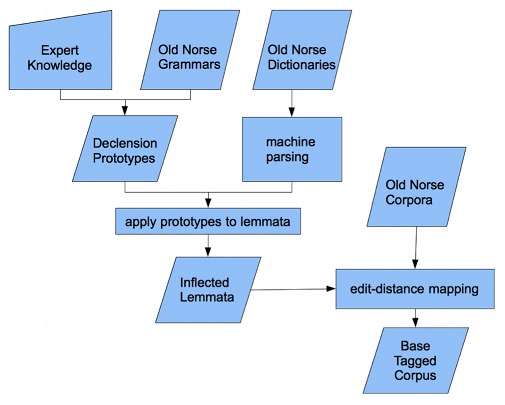
Creation of a base tagged corpus within IceMorph using various data sources. Dictionaries and corpora are machine parsed and inserted into a relational database. Declension prototypes are created by an expert via a functional programming language using readily available Old Icelandic grammars. Each dictionary lemma is mapped to corresponding declension prototypes to yield multiple declension paradigms. Finally, each corpus instance is compared to the list of inflected lemmata to produce the base tagged corpus.

**Figure 2:**
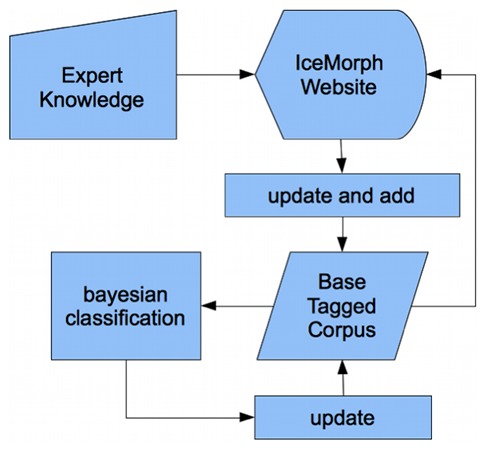
Integration of expert feedback to continuously improve POS and morphosyntactic tagging. Human experts update and enrich the existing base tagged corpus via a website interface. A machine learning algorithm continuously updates its tagging performance based on new expert input.

Figures 1 and 2 depict the general layout of IceMorph. In the following paragraphs, each module is described in more detail.

### Dictionaries

IceMorph currently uses two standard dictionaries of Old Icelandic for basic lexical and grammatical information: Cleasby-Vigfusson [3] (including the Lexicon Poeticum) and Zoëga [4]. The dictionaries were gathered from online sources [7], [8], [9] or transformed into electronic text using optical character recognition. Each dictionary entry was machine parsed and, where necessary, normalized into standard Old Icelandic orthography using the widely accepted *Íslenzk fornrit* orthographical conventions [10].

Each of the two dictionaries features approximately 27,000 entries with 42% overlap in headwords. We considered Fritzner [2] as an additional resource because it contains considerably more unique lemmata compared to Cleasby-Vigfusson or Zoëga. However, its lack of morphosyntactic detail in its entries led us to disregard it for the purposes of this study.

We encountered a number of issues during this initial data preparation phase that can be classified into three problem areas as follows:

(1) **OCR errors and other inconsistencies in underlying data:** Although OCR errors are to be expected, we have uncovered both errors and inconsistencies in each of the underlying dictionaries. We corrected a number of those errors to reduce their influence on other modules of the IceMorph system.

For instance, while Zoëga differentiates between *ø* & *ö*, æ & œ, and uses -*st* for the mediopassive forms, Cleasby-Vigfusson only uses æ, *ö*, and -*sk*. Related characters (e.g. *i* and *í*) were often interpreted incorrectly by our OCR software.

(2) **Disagreement between sources:** not all sources agree on the classification of individual lemmata. For instance, Cleasby-Vigfusson defines *báðir* as a dual adjectival pronoun (adj. pron. dual), while Zoëga lists it simply as an adjective, but considers its dual form *b*æ*ði* as a conjunction. We relied on [41] to mediate these differences.

(3) **Inconsistencies in the use of morphosyntactic information:** we relied heavily on morphosyntactic clues present in the dictionaries to determine the class of a given verb or noun. However, the same morphosyntactic syntax was often used within the same dictionary to describe lemmata belonging to different classes.

On the other hand, morphosyntactic elements of irregular forms often had unique patterns that also affected classification negatively. For instance:

**faðir (gen., dat. and acc. föður, pl. feðr)**, m. *father*.

**feðr**, m. *father*,  = faðir.

The pattern [LEMMA]+“, m.” + [TRANSLATION] usually signals masculine a-class nouns in Zoëga, so our machine parser defined a lemma *feðr*. The same dictionary contains an additional entry for *faðir* with a unique morphosyntactic structure. In this case, the machine parser was unable to categorize the lemma.

In a final step, we performed alignment on our various dictionary sources to produce a single uniform multi-dictionary relational database structure. Ambiguous or overlapping entries were discovered using simple SQL queries, and the limited number of problematic entries that we discovered were subsequently corrected by hand. Our current merged dictionary contains 48,973 lemmata. While this dictionary covers most words found in the Old Icelandic prose corpus, it has less comprehensive coverage for compounds, names, and archaic words. Each lemma is associated with at least one source entry in the dictionaries. Table 2 shows a sample source entry for lemma *afdrykkja*.

**Table 2:** Sample source entry for lemma *afdrykkja*.

**LEMMA**	afdrykkja
**COMPOUND (IF EXISTS)**	af-drykkja
**PART OF SPEECH**	noun
**CLASS (IF EXISTS)**	feminine –ijo:n
**DEFINITION/TRANSLATION**	u, f. <i>over-drinking, drunkenness,</i> = ofdrykkja [af- intens.]
**SEMANTIC EQUIVALENCES**	= ofdrykkja

Each lemma may contain a separate source entry for each dictionary source. Different source entries are linked through semantic equivalence.

Both Cleasby-Vigfusson and Zoëga contain numerous definitions referring to other lemmata, typically using symbols such as “ = ” or “cf”. For instance:

**œði-vindr (noun m_a)** = -veðr

**œði-veðr (noun n_a)** = -stormr

**œði-stormr (noun m_a)** = furious gale

We capture these semantic associations between lemmata in our source entry definitions (see Table 2 for an example). As an aside, both dictionaries contain instances of missing lemmata for a given semantic association, but those instances are fortunately rare.

### Corpora

**Figure 3:**
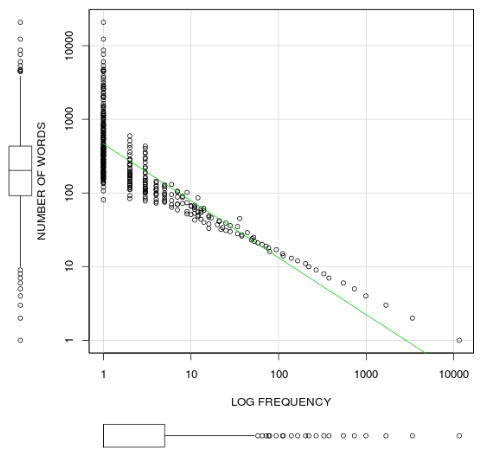
Distribution of unique word frequency in the Old Icelandic Legendary Sagas. As expected, the corpus follows a logarithmic distribution. IceMorph takes advantage of the universal fact that relatively few unique words in a corpus tend to occur with high frequency.

IceMorph uses the Icelandic Legendary Sagas [13] as a target corpus. The corpus spans a total of 357,604 non-unique words and 22,815 unique words. Figure 3 illustrates the distribution of unique word frequencies in the corpus. Its logarithmic shape confirms Zipf's law [26] that few words occur with very high frequency. We take advantage of this common property by having human experts correct paradigms of high frequency words. We also take advantage of the fact that many of these high frequency words are conjunctions as well as other words that do not inflect. The effect is a sizeable reduction in the noise related to POS and morphosyntactic information.

### Declension prototyping

IceMorph performs morphosyntactic classification in two steps. First we create declension prototypes for the most common nouns, verbs, and adjectives with the objective of creating prototypes that can generate declension paradigms for words whose inflections contain no or few irregularities. In keeping with the inherent methodology of IceMorph, we used readily available Old Icelandic grammars [4], [14] to produce those paradigms.

We integrated the declension paradigms into the system using the Functional Grammar (FM) approach [11], [12], [22], which represents an intuitive method for implementing natural language morphology in the functional language Haskell [15].

**Figure 4:**
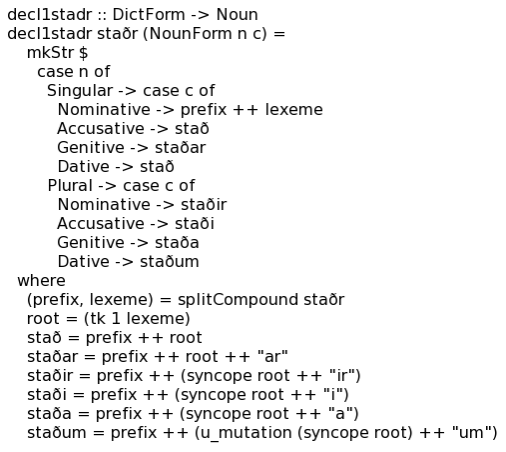
FM implementation of Old Icelandic masculine *i*-stem noun. Each declension entry is defined towards the end of the segment. Functions like ‘u_mutation’ or ‘syncope’ operate on the declension entry in question to execute the desired string manipulation.

The coding of Old Icelandic inflectional rules in FM/Haskell is accessible and easily understood by non-programmers, a necessary development criterion given the general lack of programming expertise among Old Icelandic language specialists. Such coding allowed us to take advantage of a panel of three Old Icelandic language experts who could then check for inaccuracies in the declension prototypes, which would have been impossible if we had used a different method of coding the inflection module. For instance, Figure 4 illustrates the implementation of Old Icelandic masculine *i*-stem nouns using FM. While using Old Norse “staðr” as its sample noun, this paradigm produces correct or near-correct declension paradigms for most masculine *i*-stem nouns in Old Icelandic.

IceMorph has a total of 96 prototypes: 40 noun prototypes covering nine strong and three weak declensions, 55 verb prototypes describing seven strong as well as four weak classes, and one adjective prototype. Each prototype in turn populates declension tables of varying sizes. For instance, noun declension tables consist of eight entries while verb declension tables contain 55 inflectional forms.

Using these declension prototypes, we created inflection paradigms for each lemma in our composite dictionary. Depending on the properties of a lexicon entry, we performed one of the following mappings:

*Case 1 - known morpho-syntactic classification*: If the lemma is associated with POS and class information, we generate paradigms for each prototype matching this information. For instance, lemma **af-runr** was classified as a masculine *i*-stem by the dictionary parser. There are two prototypes for masculine *i*-stem nouns, so two inflectional paradigms with a total of sixteen entries were created for this lemma.

*Case 2 - unknown class*: If, for a given lemma, the dictionary parser was only able to determine POS but not class, then inflectional paradigms were generated using each prototype of the given POS. In all cases, we were able to determine the gender of nouns and whether a verb was weak or strong. For a strong verb, such as **antigna**, we generated 20 inflectional paradigms with a total of 1100 entries.

*Case 3 - unknown classification*. For a purely hypothetical case in which neither POS nor class are known, declensions for all prototypes would be generated.

At the end of this process, IceMorph produced approximately one million declension paradigms to which we added closed-class words taken directly from our composite dictionary.

Given the Old Icelandic target corpus and the generated list of inflectional paradigms, we were able to classify each word in the corpus using the Wagner-Fischer edit distance algorithm [16]. Each unique word in the corpus was compared to the set of declensions and classified as the declension with the smallest edit distance. To reduce computational overhead, we made the following three assumptions:

1. compound prefixes do not undergo transformations; if a corpus word does not begin with the prefix of a compound word in the dictionary, the pair is skipped
2. certain Old Icelandic characters must be present in the corpus word if they are present in the lemma, and vice versa
3. the edit distance cost of transforming a declension instance into a corpus word could not exceed a value of 2

Furthermore, we used a modified cost schema tailored to the characteristics of Old Icelandic sound changes. For instance, the Old Icelandic character “a” might transform into an “ö” due to a process called *u*-mutation, so we reduced the transformation cost for those characters to a value of 0.2 (see Table 3 for more examples). On the other hand, “e” rarely changes to “ö” in Old Icelandic, so its cost remains fixed at 1. The purpose of adjusted cost is to make IceMorph less susceptible to errors, such as those generated by optical character recognition, that occur in upstream system components.

**Table 3:** Examples of edit-distance transformations and their associated cost.

TYPE OF CHANGE	FROM	TO	COST
gemination	E	t, or r	0.2
simplification	r, t, or n	E	0.2
assimilation	r	l	0.2
assimilation	ð	d, t, or s	0.2
devoicing	n, or g	k	0.2
consonant loss	l, or n	E	0.2

The transformations are specific to Old Icelandic. Their purpose is to improve classification performance by making the classifier more robust with respect to errors introduced earlier in the IceMorph system, such as OCR errors or differences in spelling convention between words in the corpus and dictionary sources.

At the end of this process, over 98% of the corpus was tagged for both POS and morphosyntactic class. Although this approach provided broad coverage, we anticipated considerable noise in these tags mainly due to the creation of imperfect declension paradigms. One of the key features of the IceMorph design is to allow expert users to manually correct data. To that end, we developed an online tool [17] that enables expert users (currently a committee of three Old Icelandic language experts) to edit and correct any data point. At the time this article was written, our experts had tagged 490 (∼0.14%) corpus words involving 289 (0.59%) dictionary entries.

Language specific phenomena such as homonymy also lead to ambiguity in classification. Homonymy is common in Old Icelandic. For instance, the corpus instance noun **menn** (“men”) could be the Nominative or Accusative Plural of the lemma **maðr**. In order to provide correct MS classification for an observed word, we needed to consider its context in the corpus. For example, a classifier is more likely to classify **menn** as Accusative Plural if it is preceded by an Accusative Plural pronoun such as **sína**. This type of context sensitive tagging is well described in the literature [27], [30], [31].

The second portion of the IceMorph system is designed to address issues related to context-based morphosyntactic (MS) tagging.

### Semi-supervised morphosyntactic (MS) classifiers

IceMorph now has two very different sources of information for POS/MS tagging. On the one hand, there are prototype-generated inflectional paradigms that operate in conjunction with the edit-distance based mapping between corpus words and declension entries. Their coverage is expansive yet very noisy. On the other hand, we have a small set of declensions contributed by our experts.

As Table 4 shows, expert feedback is considered to be correct by default. On the other end of the spectrum, prototype mappings using edit distance are expected to contain a considerable degree of noise. The two intermediate knowledge sources result from homonyms and multiple occurrences of a word in a given inflection paradigm. The table also reveals an inverse relation between the usefulness of a knowledge source and its coverage of corpus words. We refer to the first three types of feedback as “expert-related”. Combined, they provide considerable corpus coverage (∼67.6%) with relatively low noise levels.

**Table 4:** Different knowledge sources.

NAME	SOURCE	NOTE
Expert Feedback	Declension table manually entered by a language expert for a specific word in the corpus and checked for accuracy by a second expert	Assumed accurate; corpus coverage: ∼0.14%
Unique matches	Corpus words that match a single expert form	Likely accurate; corpus coverage: ∼31.9%
Non-unique matches	Corpus words that match multiple expert forms	One of the forms likely accurate; corpus coverage: ∼35.6%
Edit-distance mapping	Corpus words that do not match an expert form; by default they are mapped to one or more prototype forms with the smallest edit-distance between them	Least likely to be accurate; ∼31%

These different knowledge sources are associated with varying degrees of likelihood of providing noise-free data (overall corpus coverage: >98%).

Our classification module attempts to improve overall tagging accuracy based on this data. Our strategy was to classify MS tags directly and then infer the corresponding POS tags via simple lookup (for instance, the MS tag *nom_sg* uniquely maps to the POS tag *noun*). We considered three types of classifiers for this classification task: a dynamic Bayesian network classifier, a Hidden Markov Model (HMM) classifier with maximum likelihood estimation (MLE) using both a default and restricted Viterbi algorithm, and a linear chain Conditional Random Field (CRF) classifier.

For a given event, the **dynamic Bayesian network classifier** [20] considers its prior likelihood, as well as its likelihood in the presence of other (presumably independent) features to determine the likelihood of the event itself. The following function picks the feature set yielding maximum likelihood.
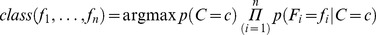
(1)In the context of IceMorph, the prior likelihood is the distribution of morphosyntactic tags based on expert feedback as well as unique and non-unique matches. The features chosen are the morphosyntactic tags preceding and following a given corpus word. We then calculate the likelihood of a given morphosyntactic element being associated with that word (Table 5).

**Table 5:** Probabilities for given target words using context feature window size = 3.

LEFT CONTEXT	TARGET WORD	RIGHT CONTEXT	PROBABILITY
dat_sg_masc	nom_sg_masc	acc_pl	0.00024
dat_sg_masc	nom_sg_masc	preposition	0.00024
dat_sg_masc	nom_sg_masc	nom_pl	0.00024
dat_sg_masc	neut_strong_pl_pos_nom	acc_pl_masc	0.00024
dat_sg_masc	neut_strong_pl_pos_nom	act_opt_pres_1_sg	0.00048
dat_sg_masc	neut_strong_pl_pos_nom	adverb	0.00024
dat_sg_masc	neut_strong_pl_pos_nom	nom_pl_neut	0.00096
dat_sg_masc	neut_strong_pl_pos_nom	conjunc	0.00143
dat_sg_masc	neut_strong_pl_pos_nom	gen_pl_masc	0.00024
dat_sg_masc	neut_strong_pl_pos_nom	acc_pl_neut	0.00096

The first three rows illustrate relatively low probabilities for unlikely POS combinations: in this example, two consecutive pronouns. The remaining rows show how more likely POS sequences receive higher probability scores; for instance, the probability of finding a word associated with MS tag *nom_sg_masc* given that it is preceded by *dat_sg_masc* and followed by *acc_pl* is 0.00024.

We restrict the knowledge sources for these features by prioritizing them from most to least strict. For instance, if a preceding word is the unique match of a given expert form, then only that morphosyntactic tag is used when calculating likelihood. If, on the other hand, it does not match any expert-based tags, then all available edit-distance tags are used.

Previous studies have shown that dynamic Bayesian network classifiers are associated with a number of attractive features, such as computational efficiency [18] as well as robustness in the presence of noisy input [19] and missing data [33], [34] due to their integration over the complete feature space. It has also been shown that these classifiers perform well even if the feature independence requirement has been violated [35].

**Hidden Markov Models** [36] are widely used for the task of sequence tagging. The HMM defines the problem space in terms of

- *S* hidden states; in IceMorph, these are morphosyntactic tags
- *O* observations; in IceMorph, these are corpus words
- transition probabilities *T_i = 1..S,j = 1..S_* between two states *i* and *j*
- emission probabilities *E_i = 1..S_* capturing the probability of an outcome for state *i*

We use a standard trigram HMM. In order to find the most likely sequence of hidden states based on given observations, we implement the Viterbi algorithm [37]. For a given t ∈ T and observations *o_1_*, …, o_n_ we find the most likely state sequence by solving
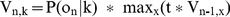
(2)for a given element *x* in the sequence.

Similar to the process applied when creating the dynamic Bayesian network classifier, we only used expert-related data from our corpus when creating the HMM. In addition, we created two versions of the Viterbi algorithm, a default and a restricted version. The default Viterbi (dV) uses all the transition probabilities offered by the HMM. In contrast, the restricted Viterbi (rV) [38] uses the expert-related subset of transition probabilities whenever they are available.

**Conditional Random Fields** [27], [32] is an undirected graphical model often used for tagging sequential data. A CRF assigns probabilities to output nodes based on the values of input nodes. In contrast to the HMM, it includes sequential knowledge and allows for the inclusion of feature functions describing the feature space. A linear-chain CRF takes into account features from the current and previous position in a given sequence and provides a score such that:
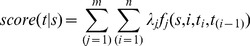
(3)for a given position *i* in a sequence of words, where *f_j_* denotes a feature function and λ_j_ represents its corresponding weight. Its feature space may include a variety of data, such as corpus instances, POS, morphosyntactic tags, positioning in a given sequence, etc. This makes CRFs quite powerful, but at a higher computational cost. Our experiments were conducted using the open source CRF++ tool [39].

## Results and Discussion

### Tagged corpora

When we started work on IceMorph we manually tagged a subset of 462 words. They were randomly chosen but reflect the relative frequency distribution of POS in Old Icelandic. We refer to this tagged set as the GOLD corpus.

In addition to the creation of GOLD, we asked our language experts to check and, if necessary, correct declension paradigms created by our prototype classifier via our online tool. At the point of writing this article 488 corpus words had been processed by our experts; we refer to this tagged set as the EXPERT corpus.

**Figure 5:**
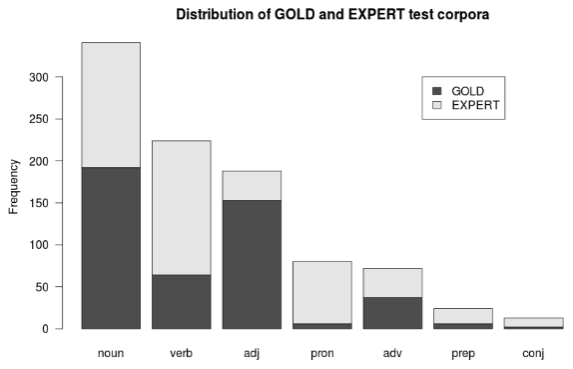
IceMorph uses two distinct test sets to evaluate classification performance. Corpus GOLD consists of 462 randomly selected corpus words. Corpus EXPERT, on the other hand, consists of 488 words tagged by expert users. This figure shows the relative frequency of POS in EXPERT and GOLD.

Figure 5 provides details with respect to the two subsets we used for testing and evaluation. The two test corpora differ in nature. Since GOLD instances have been chosen randomly they are distributed evenly throughout the corpus. In addition, words representing high frequency POS (as measured by occurrence in a dictionary) such as nouns (192 GOLD instances) and adjectives (153 GOLD instances) occur in GOLD relatively more often than words that belong to less frequent POS.

EXPERT instances, on the other hand, tend to cluster at the beginning of the corpus because our language experts focused on that section. Moreover, EXPERT contains many instances of words occurring frequently in the corpus even though the relative frequency of their associated POS in the dictionary may be lower (for instance, verbs with 160 instances or about 33%, and pronouns with 74 instances or about 15%). Table 6 shows the distribution of POS in EXPERT, GOLD, and in our concatenated dictionary.

**Table 6:** Relative distribution of POS in the IceMorph dictionary, GOLD, and EXPERT.

POS	DICTIONARY (%)	GOLD corpus (%)	EXPERT corpus (%)
noun	64.39	41.56	30.53
adjective	18.45	33.12	7.17
verb	8.45	13.85	32.79
adverb	3.93	8.01	7.17
pronoun	0.13	1.29	15.16
preposition	0.1	1.29	3.69
other	4.54	0.88	1.24

The tagged corpus GOLD resembles more closely the distribution of the dictionary while the tagged corpus EXPERT owes its pattern of distribution to frequencies in the saga corpus.

When testing classifiers we distinguish between results obtained using EXPERT and GOLD, respectively. EXPERT is our closest analogy to a properly tagged test environment because it contains long sequences of tagged words. GOLD, on the other hand, allows us to study the robustness of a given classifier since most of its instances occur in a highly noisy environment (i.e. preceding and following words tend to not be tagged).

The data used for this project is available through the California Digital Library's “Merritt” data repository. We have deposited three sets of data in the repository which can be used in conjunction with our code, available from GitHub. The three datasets are collected as a single data package on Merritt, with the following DOI: 10.5068/D1WC7K. The contents of this package is as follows:

- the concatenated dictionary file, stored as a json (dictionary_20140605.json)
- the untagged and tagged Fornaldarsögur corpus (allvol.zip and icemorph_corpus-2014-06-01.zip)
- the EXPERT and GOLD training/testing corpora (tagged_corpora_20140605.json)

### Classification results

As a baseline measure, we ran all classifiers on an in-sample data set (i.e., the same data was used for training and testing) for both the EXPERT and GOLD tagged sets. As expected, all classifiers performed well. We then split our test data into 80% training and 20% testing. In future work, the selection of corpus instances will be driven by “Query by Uncertainty”, an active learning algorithm that [40] has shown to provide increased accuracy for corpora with minimal training sets. From the EXPERT corpus we used the first 20% for testing because forms tagged by experts tend to be clustered around the beginning of our corpus. Since the GOLD forms are more evenly spread throughout the corpus, we chose the last 20% as test data.

When applying our classifiers to the split data set, the HMM classifier clearly outperformed the other two, its accuracy not suffering relative to its baseline (indeed, it scored higher). The restricted Viterbi consistently performed superior relative to the default Viterbi. This is pronounced in the performance of HMM-rV on the GOLD corpus, which contains a higher degree of uncertainty. With respect to results from EXPERT corpus on the POS tagging task, our HMM classifier yields results similar to state-of-the-art POS taggers trained on noise-free data. Table 7 contains the results of our classification tests.

**Table 7:** Accuracies for POS and MS tagging.

TEST	POS EXPERT	POS GOLD	MS EXPERT	MS GOLD
Bayes-base	95.43%	79.25%	80.67%	48.34%
Bayes-80/20	85.71%	75.14%	62.37%	43.24%
HMM-dV-base	93.85%	25.60%	75.82%	13.62%
HMM-dV-80/20	93.68%	34.74%	82.11%	18.75%
HMM-rV-base	96.11%	71.58%	79.92%	53.98%
HMM-rV-80/20	96.84%	73.16%	84.21%	54.86%
CRF-1-base	89.75%	36.58%	78.07%	11.54%
CRF-1-80/20	87.30%	46.07%	77.78%	16.55%
CRF-2-80/20	84.13%	48.69%	56.08%	17.24%

Tests with postfix “base” were performed using in-sample test sets. For the others, the supervised set was split into 80% training and 20% testing.

The relatively poor performance of the CRF classifier deserves special explanation. Due to its higher demand for computing resources, we initially restricted its training set to sequences in which each word was associated with no more than one morphosyntactic form. As features we chose surface forms and MS tags of the preceding and following corpus words. Test CRF-1-80/20 performed below its in-sample base line, but the decline was considerably less than the dynamic Bayesian network classifier. We assumed that increasing the number of allowed morphosyntactic forms associated with a given word from one to two we could improve CRF performance. But as test CRF-2-80/20 shows, the opposite was true: performance declined somewhat for EXPERT words. Our interpretation of these results is that while CRF performs very well when trained with noise-free input, it is less capable of handling uncertainty in its training set than our HMM classifier with restricted Viterbi.

## Conclusion and Outlook

The IceMorph POS and MS tagger attempts to maximize classification performance using a minimum of cleanly tagged training data. It is a hybrid system combining readily available resources for Old Icelandic (such as dictionaries, grammars, and corpora) and human expert feedback with machine learning algorithms for continuous automated classification. Given a small set of tagged words, IceMorph achieves corpus-wide POS classification of over 96% and MS classification of over 84% accuracy.

None of the resources used by IceMorph is noise free. Dictionaries and corpora contain errors introduced during OCR or inherent in the source itself. Furthermore, the context-based classifier learns its probability matrix from highly noisy data. IceMorph is designed to maximize performance given this noisy environment. It does so by taking cues from human experts, as well as exploiting the logarithmic distribution of unique words in corpora, essentially reducing the task of classification to a process of disambiguation of homographs.

The key to improved performance will be to further reduce noise throughout the IceMorph system, most easily accomplished by expanding expert feedback. We are exploring additional ways to improve accuracy by refining our machine learning algorithms. We are also investigating how to optimize the selection of corpus words to have maximum impact on classification performance by implementing appropriate active learning algorithms. Finally, we are looking at ways to incorporate phenomena specific to Old Icelandic, such as enclitics (suffixed determiners), so as to reduce classification failures.

## Software and Data

Software for this project can be found at GitHub. Search for IceMorph. Data is available at the University of California/California Digital Library repository Merritt, with the following DOI: 10.5068/D1WC7K
